# Quantitative SPECT/CT imaging of actinium-225 for targeted alpha therapy of glioblastomas

**DOI:** 10.1186/s40658-024-00635-1

**Published:** 2024-05-09

**Authors:** Monika Tulik, Radosław Kuliński, Zbisław Tabor, Beata Brzozowska, Piotr Łaba, Frank Bruchertseifer, Alfred Morgenstern, Leszek Królicki, Jolanta Kunikowska

**Affiliations:** 1 Department of Nuclear Medicine, Medical University of Warsaw, Warsaw, Poland; 2 Faculty of Electrical Engineering, Computer Science, and Biomedical Engineering, AGH University of Science and Technology, Krakow, Poland; 3 Biomedical Physics Division, Faculty of Physics, University of Warsaw, Warsaw, Poland; 4 Joint Research Centre, European Commission, Karlsruhe, Germany

**Keywords:** Quantitative imaging, SPECT/CT, Dosimetry, Actinium-225, Glioblastoma

## Abstract

**Background:**

A new, alternative option for patients with recurrent glioblastoma is targeted alpha therapy (TAT), in the form of a local administration of substance P (neurokinin type 1 receptor ligand, NK-1) labelled with ^225^Ac. The purpose of the study was to confirm the feasibility of quantitative SPECT imaging of ^225^Ac, in a model reproducing specific conditions of TAT. In particular, to present the SPECT calibration methodology used, as well as the results of validation measurements and their accuracy. Additionally, to discuss the specific problems related to high noise in the presented case.

**Materials and methods:**

All SPECT/CT scans were conducted using the Symbia T6 equipped with HE collimators, and acquired with multiple energy windows (three main windows: 440 keV, 218 keV, and 78 keV, with three lower scatter energy windows). A Jaszczak phantom with fillable cylindrical sources of various sizes was used to investigate quantitative SPECT/CT imaging characteristics. The planar sensitivity of the camera, an imaging calibration factor, and recovery coefficients were determined. Additionally, the 3D printed model of the glioblastoma tumour was developed and imaged to evaluate the accuracy of the proposed protocol.

**Results:**

Using the imaging calibration factor and recovery coefficients obtained with the Jaszczak phantom, we were able to quantify the activity in a 3D-printed model of a glioblastoma tumour with uncertainty of no more than 10% and satisfying accuracy.

**Conclusions:**

It is feasible to perform quantitative ^225^Ac SPECT/CT imaging. However, there are still many more challenges that should be considered for further research on this topic (among others: accurate determination of ICF in the case of high background noise, better method of background estimation for recovery coefficient calculations, other methods for scatter correction than the dual-energy window scatter-compensation method used in this study).

## Background

Glioblastomas are the most common primary brain tumour with a poor prognosis [1]. Approximately 40% of patients survive the first year after diagnosis and 17% in the following year. Due to its infiltrative nature, molecular heterogeneity, and only partially preserved function of the blood-brain barrier, the median overall survival time of these patients is 9–15 months, regardless of comprehensive treatment including surgery, external beam radiotherapy, and chemotherapy [2]. Despite the search for new treatment methods, recurrence of the disease is observed in 90% of patients within 6 months. There has been no significant improvement in the treatment of this disease in recent decades. A new, alternative option for patients with recurrent glioblastoma is the targeted alpha therapy (TAT), in the form of a local administration of substance P (neurokinin type 1 receptor ligand, NK-1) labelled with isotopes that undergo alpha decay, ^213^Bi or ^225^Ac [3]. Many advantages of using substance P over other vectors in this case have been demonstrated [4]. In addition, alpha particles have a definite advantage over other types of ionizing radiation generated as a result of radioactive decay, related to their physical properties (the short ranges in the tissue and the high linear energy transfer). Ultimately, in TAT, a significant therapeutic effect should be achieved, while minimizing side effects on surrounding healthy tissues. In phase I and II studies initiated by researchers from the Medical University of Warsaw, local injection of substance P labelled with ^213^Bi/^225^Ac in the case of patients with relapsed glioblastoma provides satisfactory results with prolongation of survival parameters compared to standard treatment [3, 5, 6]. So far, the dosimetry of the target volume was not performed and the achieved therapeutic effect was not correlated with the delivered absorbed dose. However, the researchers found that the therapeutic effects was independent of the isotope (the same results for ^213^Bi or ^225^Ac). The experience to date shows strong evidence, that local radioisotope treatment of brain tumours requires dosimetry studies, taking into account the complexity of biological processes [6].

**Fig. 1:**
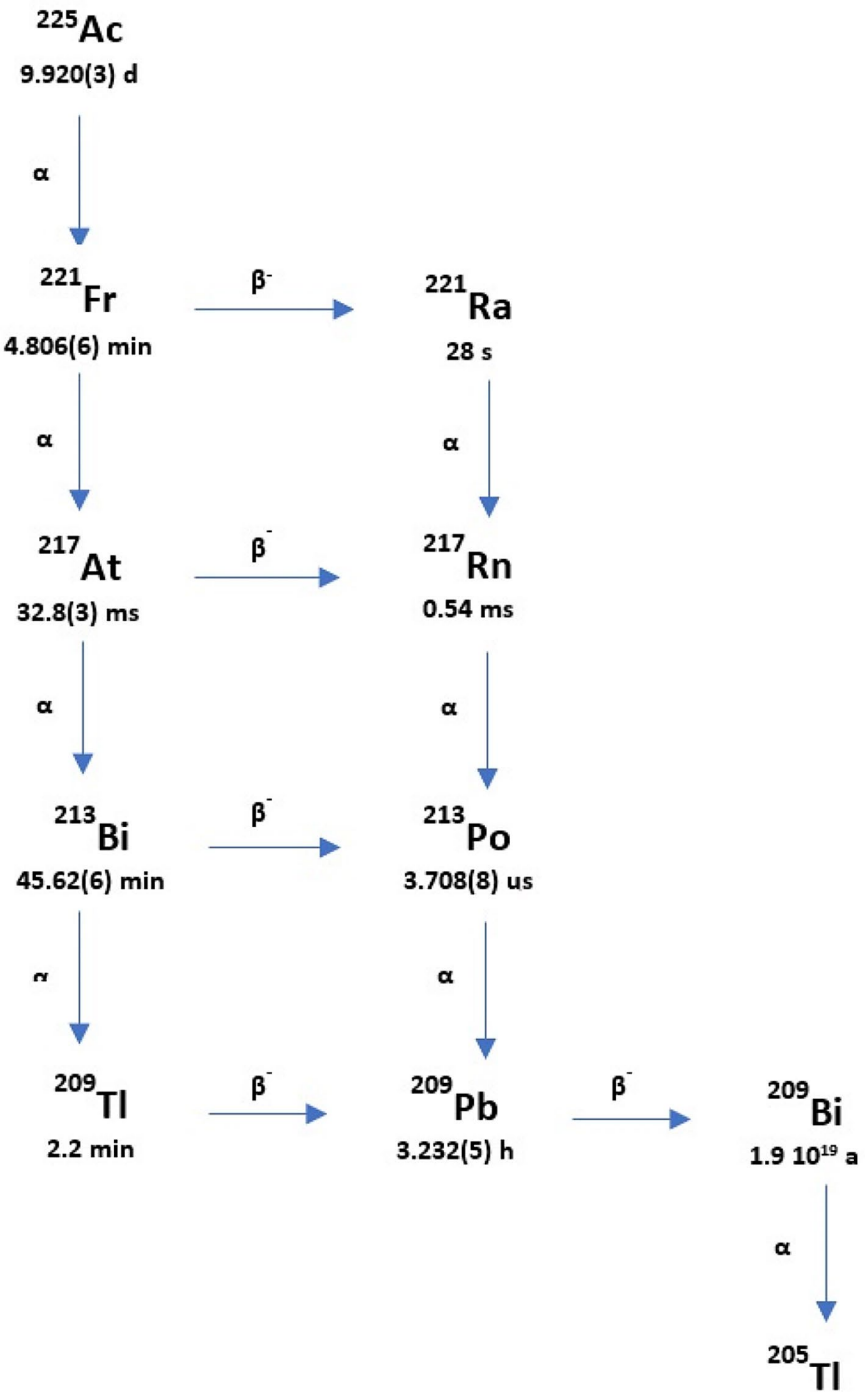
The schematic decay chain of ^225^Ac (based on Suliman G. et al. [7])

The first step in calculating the absorbed dose in the case of TAT (as in other forms of radionuclide therapies) is the quantitative imaging of the radiopharmaceutical distribution in the patient’s body. The physical characteristics of ^225^Ac emissions results in a complicated decay scheme (Fig. 1). Finally, from the point of view of the energy spectrum registered by the gamma camera, two principal gamma emissions at 218 keV and 440 keV (from its daughters ^221^Fr and ^213^Bi, respectively), along with bremsstrahlung X-rays from ^209^Pb and many scattering photons, are observed. It appears that the quantitative imaging of ^225^Ac could be even more challenging compared to other therapeutic radionuclides (i.e. emitting beta-particles).

Few studies refer to qualitative and quantitative imaging after administration of ^225^Ac. Kratochwil et al. acquired the first posttherapy [^225^Ac]Ac-PSMA-617 whole-body scans using the 440 keV γ-coemission of ^213^Bi, the 218 keV γ-coemission of ^221^Fr, and the bremsstrahlung of ^209^Pb with a gamma camera equipped with high energy (HE) collimators [8]. Usmani et al. and Vatsa et al. presented posttherapy whole-body scans after an administration of [^225^Ac]Ac-PSMA-617, acquired using three main photopeaks (78 keV, 218 keV, and 440 keV) and demonstrated the tracer’s distribution [9, 10]. The authors concluded that the addition of 78 keV photopeak provides better quality images, higher count statistics, and more lesion delineations. Therefore, they suggested that posttherapy imaging of [^225^Ac]Ac-PSMA-617 should be performed using three main abundant photopeaks. Ocak et al. and Kamaleshwaran et al. acquired the first SPECT/CT posttherapy scans after administration of [^225^Ac]Ac-DOTATATE, using 218 keV and 440 keV gamma rays, and HE collimators [11, 12]. Gosewisch A et al. published a short paper about the first effort to perform quantitative SPECT/CT imaging and dosimetry for [^225^Ac]Ac-PSMA-I&T [13]. Additionally, Kratochwil C et al. and Belli ML et al. performed dosimetry estimates for [^225^Ac]Ac-PSMA-617, but calculated based on time-activity-curves derived from serially performed [^177^Lu]Lu-PSMA-617 scans extrapolated to the physical half-life of ^225^Ac and taking into account their different relative biological effectiveness [14, 15]. Delker A et al. demonstrate the clinical feasibility of quantitative low-count SPECT imaging for [^225^Ac]Ac-PSMA-I&T therapy of advanced prostate cancer based on the ^213^Bi peak and provide a first image-based estimate of the radiation absorbed dose in critical organs and lesions [16]. To the knowledge of the authors, only one, comprehensive experiment investigating the capabilities to quantify SPECT of ^225^Ac was described and published [17]. Benabdallah N et al. established that three-energy emission windows are recommended in order to provide higher count statistics and to improve the quality of ^225^Ac SPECT images. According to these authors, such an acquisition protocol with multiply energy windows could be also the basis for its quantification.

The purpose of our study was to confirm the feasibility of quantitative SPECT imaging of ^225^Ac with multiple energy windows, in a model reproducing specific conditions of TAT using substance P labelled with ^225^Ac in the case of patients with recurrent glioblastoma. In particular, we present the SPECT calibration method used, as well as the results of the validation measurements and their accuracy. Additionally, to discuss the specific problems related to high noise in the presented case.

## Materials

### Gamma camera

All measurements were conducted using the Symbia T6 (Siemens Healthineers, Germany), equipped with a dual-head SPECT gamma camera (3/8” NaI(Tl) crystal, 59 photomultiplier tubes, and 53.3 × 38.7 cm field of view) and multidetector CT (6 rows). The SPECT/CT scanner was installed at the Department of Nuclear Medicine Medical University of Warsaw in 2012.

All SPECT/CT acquisitions were performed with HE collimators and with energy windows determined from energy spectrum evaluation (see Sect. 3.2).

### Phantoms

**Fig. 2:**
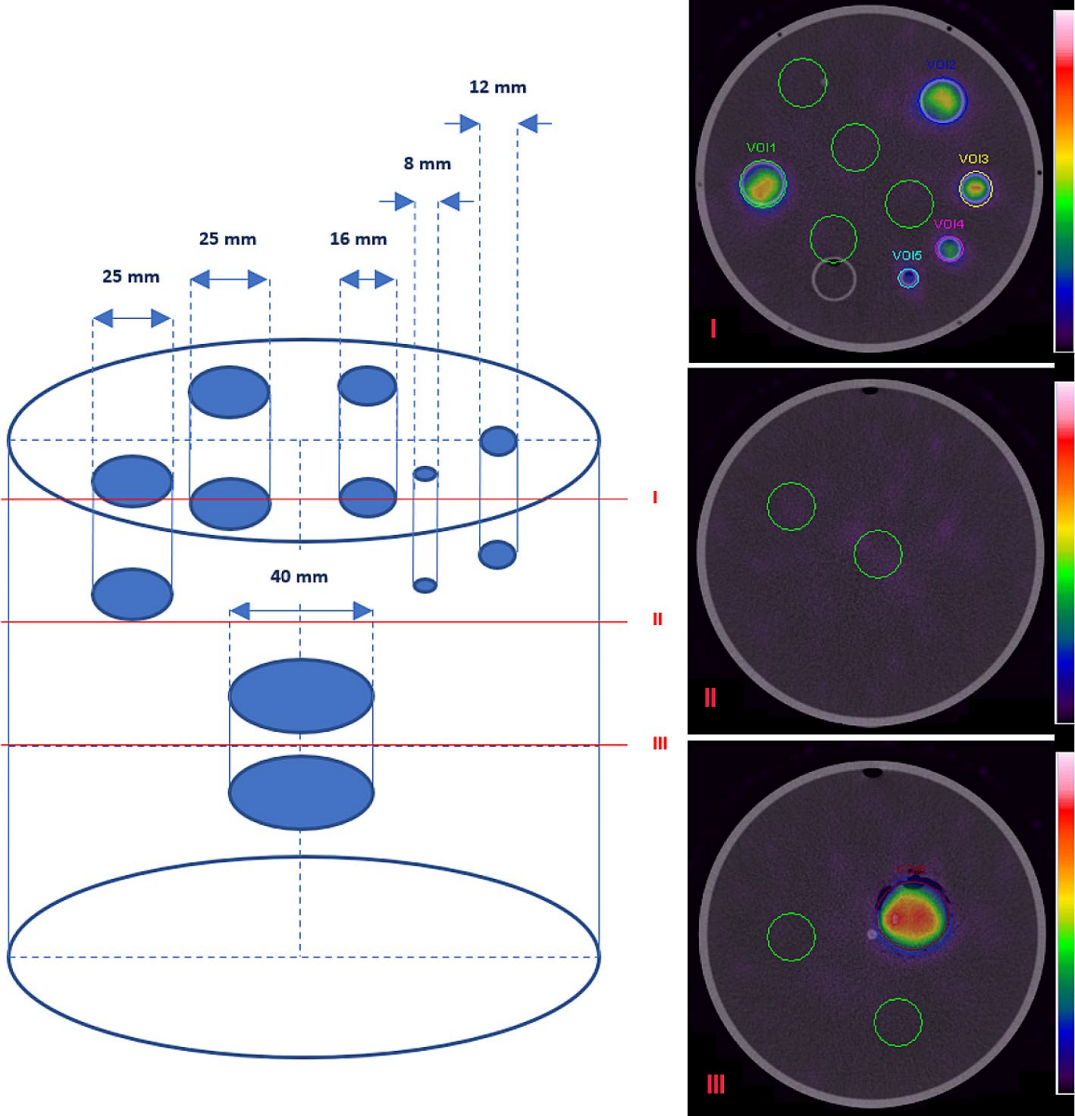
A scheme of the phantom with the positions of cylinders used as a “hot” source and their dimensions. SPECT/CT images of the phantom in various cross-sections (I, II, and III)

In our study, a body of the Jaszczak phantom with fillable cylindrical sources of various sizes (the base of the Jaszczak Deluxe Flangeless ECT phantom with a PET faceplate meeting the American College of Radiology requirements) was used to investigate quantitative SPECT/CT imaging characteristics. Although, in most quantitative imaging experiments the NEMA IEC PET Body Phantom is widely used, a chosen phantom represents better the head of the patient. The phantom body (a main cylinder) is a flangeless source thank with a 6.4 mm wall thickness, 20.4 cm interior diameter, and 18.6 cm interior height (volume 6.1 L). Both solid spheres and the rod insert were removed from the main cylinder. The faceplate has fillable thin-walled cylinders (8, 12, 16, and 3 × 25 mm in diameter with an interior height of 38 mm), and a removable Teflon cylinder. The volumes of thin-walled cylinders range from 1.9 to 18.6 mL. Only five fillable cylinders were used in the study (with diameters: 8, 12, 16, and 2 × 25 mm, respectively). A third 25 mm diameter fillable cylinder was filled with non-radioactive water. The Teflon cylinder was removed from a phantom. An additional cylindrical source with 40 mm diameter and 21 mm height (26 mL volume) was prepared homemade and positioned near the central axis of a main cylinder, below the rest of the sources. A schematic presentation of the used sources’ arrangement in the phantom, also with the phantom SPECT/CT images, are shown in Fig. 2; a location of volumes of interest (VOIs) for analysis are indicated, along with the background VOIs.

Additionally, to establish the required energy window settings and a gamma camera planar sensitivity before main phantom scanning, a solution of ^225^Ac within a plastic Petri dish (9 cm in diameter) was prepared.

### Activity concentration

^225^Ac was obtained by radiochemical extraction from ^229^Th sources and was supplied by the Directorate for Nuclear Safety and Security of the Joint Research Centre of the European Commission in Karlsruhe, Germany [18]. The sample of ^225^Ac solution used for measurements was quality controlled by high-resolution gamma spectroscopy to verify its radiological properties and purity. The final activity of ^225^Ac used for the study was measured with the CRC-15R ionization chamber radionuclide dose calibrator (Capintec, Inc., USA), installed in the Department of Nuclear Medicine Medical University of Warsaw. Its accuracy, stability (reproducibility), and linearity were verified for ^225^Ac measurements. In particular, for ^225^Ac activity measurements, an uncertainty of less than 2% was found.

The previously published paper reported the median viable tumour volume measured in magnetic resonance imaging (defined on contrast-enhanced T1 image) in the group of patients treated with [^225^Ac]Ac-DOTA-substance P as 35.7 mL [5]. The local usage of [^225^Ac]Ac-DOTA-substance P in glioblastoma patients was assessed with a minimum activity of 10 MBq. Taking the above into account, the activity concentration in this case was around 280 kBq/mL. Based on that report, an activity concentration reflecting the treatment conditions of glioblastoma patients was prepared for phantom experiments. The cylindrical sources of the phantom (hot sources that simulate the accumulation of the main pool of activity in the tumours) were filled with a similar activity concentration of the ^225^Ac solution: 270 kBq/mL. The phantom body was filled with non-radioactive water (a cold background simulating no activity in a patient’s head outside the tumour). The total activity of ^225^Ac in the phantom was (21.0 ± 0.5) MBq. However, due to technical reasons, the first scan of the phantom was not performed immediately after filling. The activity concentration in the cylindrical sources at the time of the first scan was 235 kBq/mL.

A Petri dish was filled with (3.3 ± 0.1) MBq of ^225^Ac solution.

## Methods

### Energy spectrum and planar sensitivity measurements

The Petri dish was placed in the middle of the field of view between the two gamma camera heads at a point 15 cm from both heads. The Symbia T6 directly measures the energy spectrum without acquiring an image. The energy spectrum of ^225^Ac was registered for 10 min. The same geometry and source were used to obtain a gamma camera planar sensitivity. A planar acquisition was acquired for 10 min. The planar sensitivity *S* was calculated in each main energy window as follows:

1 $$S=\frac{{Counts}_{ROI}}{A\cdot T}$$

where *Counts*_*RO*I_ denotes the total counts measured in a region of interest (ROI) with the same area as the Petri Dish (a circle ROI of 9 cm diameter), after background subtraction, *A* denotes the nominal activity in the source at the time of acquisition given in MBq, and *T* is the acquisition duration given in s.

The standard uncertainty of *S* (*u(S)*) was calculated according to the equation and the multiplicative variant of the law of propagation of uncertainty [19]:

2 $$u\left(S\right)=S\cdot \sqrt{{\left(\frac{u\left({Counts}_{ROI}\right)}{{Counts}_{ROI}}\right)}^{2}+{\left(\frac{u\left(A\right)}{A}\right)}^{2}+{\left(\frac{u\left(T\right)}{T}\right)}^{2}}$$

The standard uncertainty in the counts *u(Counts*_*ROI*_*)* within a ROI was calculated as the square root of the number of counts. The standard uncertainty on activity *u(A)* measurements was equal to 2% and for acquisition duration *u(T)* was assumed to be 1 s.

### SPECT/CT acquisition and reconstruction

Primary and scatter energy windows for ^225^Ac quantitative imaging were chosen based on the acquired energy spectrum (Table 1).

**Table 1:** Primary and scatter energy windows for ^225^Ac quantitative imaging

Windows	Primary	Lower Scatter
I	78 keV, width 20%, 70.2 keV to 85.8 keV	10% width, 62.4 keV to 70.2 keV
II	217 keV, width 10%, 206.2 keV to 227.9 keV	5% width, 195.3 keV to 206.2 keV
III	444 keV, width 10%, 421.8 keV to 466.2 keV	5% width, 399.6 keV to 421.8 keV

The phantom was placed in the centre of the field of view of the gamma camera. The parameters of the SPECT acquisition protocol are presented in Table 2. A CT scan was acquired for attenuation correction (AC), using a standard low-dose protocol.

**Table 2:** The SPECT acquisition protocol with reconstruction parameters

Collimators	HE
Orbit type and Mode	Body Contour and Step-and-Shoot
Matrix	128 × 128
Voxel size [mm]	4.8 × 4.8 × 4.8
Number of projections	64 per detector (128 projection acquired over 360°)
Time per projection [s]	30
Reconstruction	OSEM Flash 3D
Number of iterations	10
Number of subsets	8
Attenuation Correction (AC)	CT
Scatter Correction (SC)	DEW (dual energy window method)
Filtration	Gauss filter (FWHM = 4 mm)

The acquired data with multiple energy windows were reconstructed on the Esoft workstation (Siemens Healthineers, Germany) with the following parameters: OSEM FLASH 3D algorithm, 10 iterations, and 8 subsets, with Gauss filter (FWHM = 4 mm). Attenuation correction was performed using the CT-based attenuation map with the manufacturer’s default parameters. For each main energy window the user had to indicate the appropriate correction map for AC correction. An appropriate correction map was chosen by selecting one of the radioisotopes defined in the software (which emitted radiation with energy closest to a selected energy windows). In particular, for I energy window ^133^Xe was chosen (scale attenuation coefficients: 80 keV), for II energy window ^111^In was chosen (scale attenuation coefficient: 247 keV), and for III energy window ^18^F was chosen (scale attenuation coefficients: 511 keV). It can be estimated that the use of selected correction maps has a small impact on attenuation correction accuracy (around 5%, taking into account the difference between linear attenuation coefficients for water in considered energy range). The projections were corrected for scatter using the corresponding scatter windows and the dual-energy window (DEW) scatter-compensation method (SC). This method for SC was used (not the triple energy window scatter-compensation method (TEW)), because during acquisition on the Symbia T6 three main energy windows were established. Since an acquisition protocol allows to define a maximum of six independent energy windows, for three main windows only three lower scatter energy windows could be defined. The reconstructed images for individual main energy windows were summed up after reconstruction before further analysis.

The phantom was scanned according to the above protocol 9 times during 2.5 months (at a medium time of interval 8 days), starting from an activity concentration of 235 kBq/mL (a total activity in the phantom equal to 18.3 MBq) and ending with an activity concentration of 1.5 kBq/mL (a total activity in the phantom equal to 0.1 MBq).

### Image calibration factor (ICF) for ^225^Ac

All reconstructed images were evaluated using the Esoft workstation (Siemens Healthineers, Germany) volumetric analysis application. Volumes of interests (VOIs) defined on the CT image were applied to the SPECT reconstructed data. The total numbers of counts summed over the phantom reconstructed image (a VOI defined about the CT image of the outer boundaries of the phantom) were used to determine the image calibration factor (ICF). The ICF given in cps/MBq was calculated for the entire volume of the phantom using the formula:

3 $$ICF=\frac{{Counts}_{Phantom}}{A\cdot T}$$

where *Counts*_*Phantom*_ denotes the total number of counts in a VOI defined about the CT image of the outer boundaries of the phantom, after background subtraction, *A* is the activity present in the phantom at the time of acquisition given in MBq, and *T* denotes the acquisition duration given in s (*T* = 3840 s, constant for all acquisitions). The counts per mL in the background was calculated by placing eight spherical VOIs randomly in the main cylinder image (also between cylindrical sources at their height in the phantom; see Fig. 2). The standard uncertainty of the ICF (*u(ICF)*) was calculated as follows:

4 $$u\left(ICF\right)=ICF\cdot \sqrt{{\left(\frac{u\left({Counts}_{Phantom}\right)}{{Counts}_{Phantom}}\right)}^{2}+{\left(\frac{u\left(A\right)}{A}\right)}^{2}+{\left(\frac{u\left(T\right)}{T}\right)}^{2}}$$

The standard uncertainty in the counts *u(Counts*_*Phantom*_*)*, as well as on measured activity *u(A)* and for acquisition duration *u(T)* were defined as the standard uncertainty of planar sensitivity calculations.

### Recovery coefficients (RCs)

In particular, VOIs were manually defined about the physical boundary of each cylindrical source on the CT image. These CT-based VOIs were copied to the SPECT image. RCs were calculated according to the formula:

5 $$RC=\frac{{Counts}_{Cylinder}}{ICF\cdot {A}_{cylinder}\cdot T}$$

where *Counts*_*Cylinder*_ denotes the total reconstructed counts within the target VOI, after background subtraction, *A*_*Cylinder*_ is the activity present in each cylinder at the time of acquisition given in MBq, and *T* denotes the acquisition duration given in s. To summarize, RCs were calculated by dividing the SPECT-based activity in each of the cylindrical sources of nominal volume by the activity dispensed to the cylindrical source known from the phantom experiment preparation. The recovery coefficients were fitted to a three-parameter model using the following equation:

6 $$RC\left(V\right)=\frac{\gamma }{1+{\left(\frac{\alpha }{V}\right)}^{\beta }}$$

where *V* is each cylindrical source volume given in mL and *α*, *β*, and *γ* are the fit parameters. To fit RC vs. V, as given in Eq. (6), a curve-fit function from scipy python library was used. This function returns both fitted parameters: *α*, *β* and *γ* of the model, and their standard errors (*SE*). Based on residual values mean squared error was calculated from which the confidence interval for the fitted curve was obtained.

The uncertainty of RC(V) (*u(RC(V)*) was calculated following Eq. (7), under the assumption of spherical sources and the definition of target VOI on SPECT imaging [20]:

7 $$u\left(RC\left(V\right)\right)=RC\sqrt{{\left(\frac{\partial RC\left(V\right)}{\partial \alpha }\right)}^{2}{u\left(\alpha \right)}^{2}+{\left(\frac{\partial RC\left(V\right)}{\partial \beta }\right)}^{2}{u\left(\beta \right)}^{2}+{\left(\frac{\partial RC\left(V\right)}{\partial \gamma }\right)}^{2}{u\left(\gamma \right)}^{2}+{\left(\frac{\partial RC\left(V\right)}{\partial V}\right)}^{2}{u\left(V\right)}^{2}}$$

### Assessment of quantitative accuracy

**Fig. 3:**
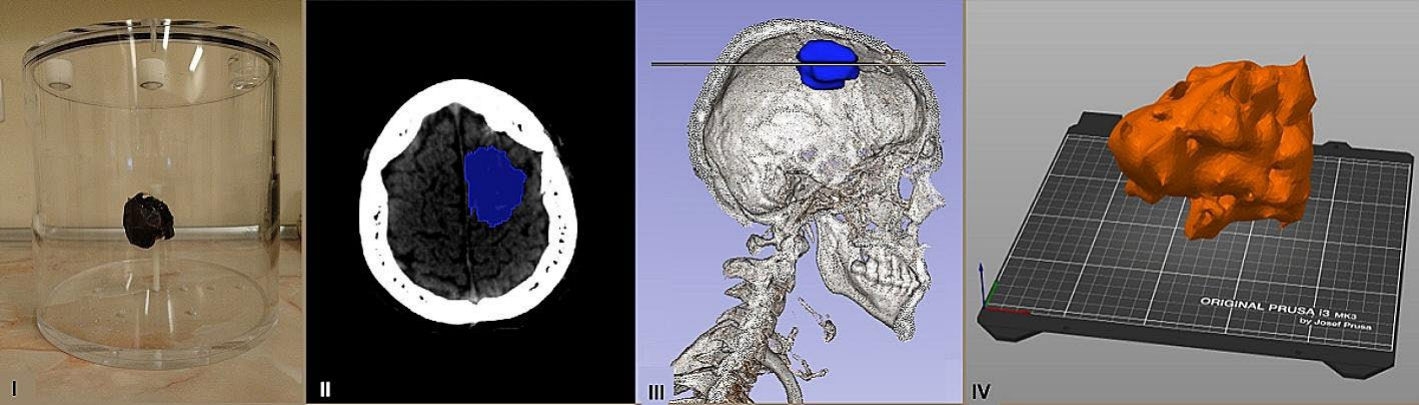
The 3D printed model and its positions in the main cylinder (I and IV). The 3D printed model was created based on CT scan: clinical target volume (II). 3D printed model located in the patient’s brain (III). The line in (III) represents the CT scan shown in (II)

An experiment was performed to validate the accuracy of the defined protocol and quantitative imaging procedure. A three-dimensional printed model of glioblastoma was prepared. The model was designed based on real CT images of the patient with a brain tumour. In collaboration with the Faculty of Physics, University of Warsaw, the stl format file was created in Slicer 3D and the 3D phantom was printed on Prusa MK3 3D printer with polylactic acid (PLA). The volume of the 3D model was 25 mL. The printed model was checked for liquate before filling with a ^225^Ac solution. Finally, it was filled with 250 kBq/mL of ^225^Ac. The total activity in the 3D model was (6.1 ± 0.1) MBq. The filled model was placed in the middle of the mail cylinder, while other inserts were removed from it (Fig. 3).

The phantom was scanned just after preparation. A SPECT/CT acquisition of the phantom was acquired with the same acquisition parameters described above on the Symbia T6 gamma camera with HE collimators. The same reconstruction parameters were then applied to obtain the final image. A VOI was contoured in the CT image of the 3D model, and the total counts inside the boundaries of that VOI in the SPECT image were determined. Additionally, eight background VOIs were defined. The mean counts per mL in the background was calculated. Taking into account the 3D model volume, the background counts were subtracted from the total counts in the 3D model VOI. The activity in the model (*A*_*3DPhantom*_) was estimated using the imaging calibration factor and the recovery coefficient obtained from experiments with the phantom and compared with the actual activity. The activity in the 3D model was calculated with the equation:

8 $${A}_{3DPhantom}=\frac{{Counts}_{3DPhantom}}{ICF\cdot RC\left(V\right)\cdot T}$$

where *Counts*_*3DPhantom*_ denotes the total reconstructed counts within the 3D model VOI, after background subtraction, *ICF* is the calculated imaging calibration factor, *RC(V)* denoted the recovery coefficient calculated according to Eq. 6, and *T* is the acquisition duration given in s.

The standard uncertainty of *A*_*3DPhantom*_ (*u(A*_*3DPhantom*_) was calculated as follows:

9 $$u\left({A}_{3DPhantom}\right)={A}_{3DPhantom}\cdot \sqrt{{\left(\frac{u\left({Counts}_{3DPhantom}\right)}{{Counts}_{3DPhantom}}\right)}^{2}+{\left(\frac{u\left(ICF\right)}{ICF}\right)}^{2}+{\left(\frac{u\left(RC(V\right))}{RC\left(V\right)}\right)}^{2}+{\left(\frac{u\left(T\right)}{T}\right)}^{2}}$$

The standard uncertainty in the counts *u(Counts3D*_*Phantom*_*)*, as well as for acquisition duration *u(T)*, were defined as above. The standard uncertainty of ICF (*u(ICF*)) was assumed to be 1,8 cps/MBq (see Sect. 4). The uncertainty in recovery at a given volume was calculated following the law of propagation of uncertainties, under the assumption of spherical sources and the definition of target VOIs in SPECT imaging [20].

The percentage difference (*%D*) between the SPECT-measured activity *A*_*3DPhantom*_, and the activity measured using the radionuclide dose calibrator (the actual activity A equal to (6.1 ± 0.1) MBq), was used to evaluate the quantitative accuracy:

10 $$\%D=100\cdot \frac{{A}_{3DPhantom}-A}{A}$$

## Results

**Fig. 4:**
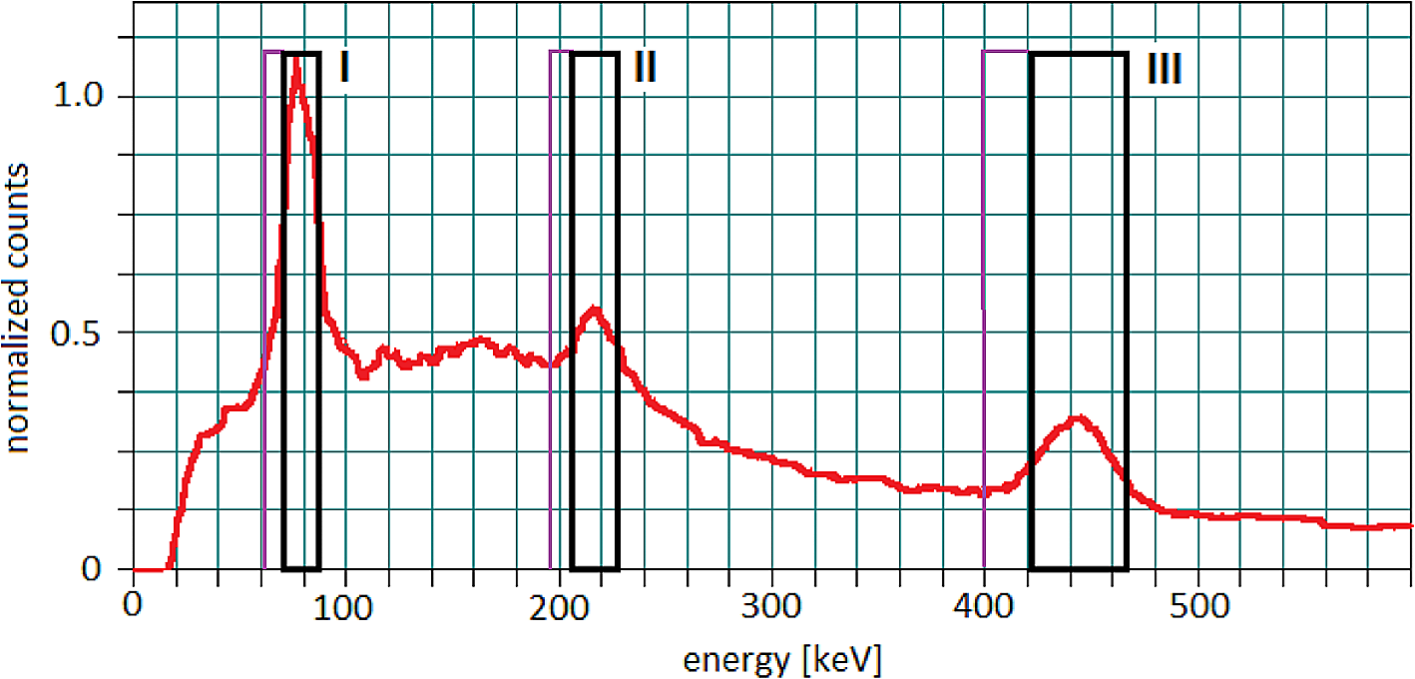
The energy spectrum was measured on the Symbia T6 with HE collimators, and chosen main energy windows (I: 78 keV, width 20%, II: 217 keV, width 10%, III: 444 keV, width 10%), with their corresponding lower scatter windows

The obtained energy spectrum, and selected emission energy windows, were presented in Fig. 4.

**Fig. 5:**
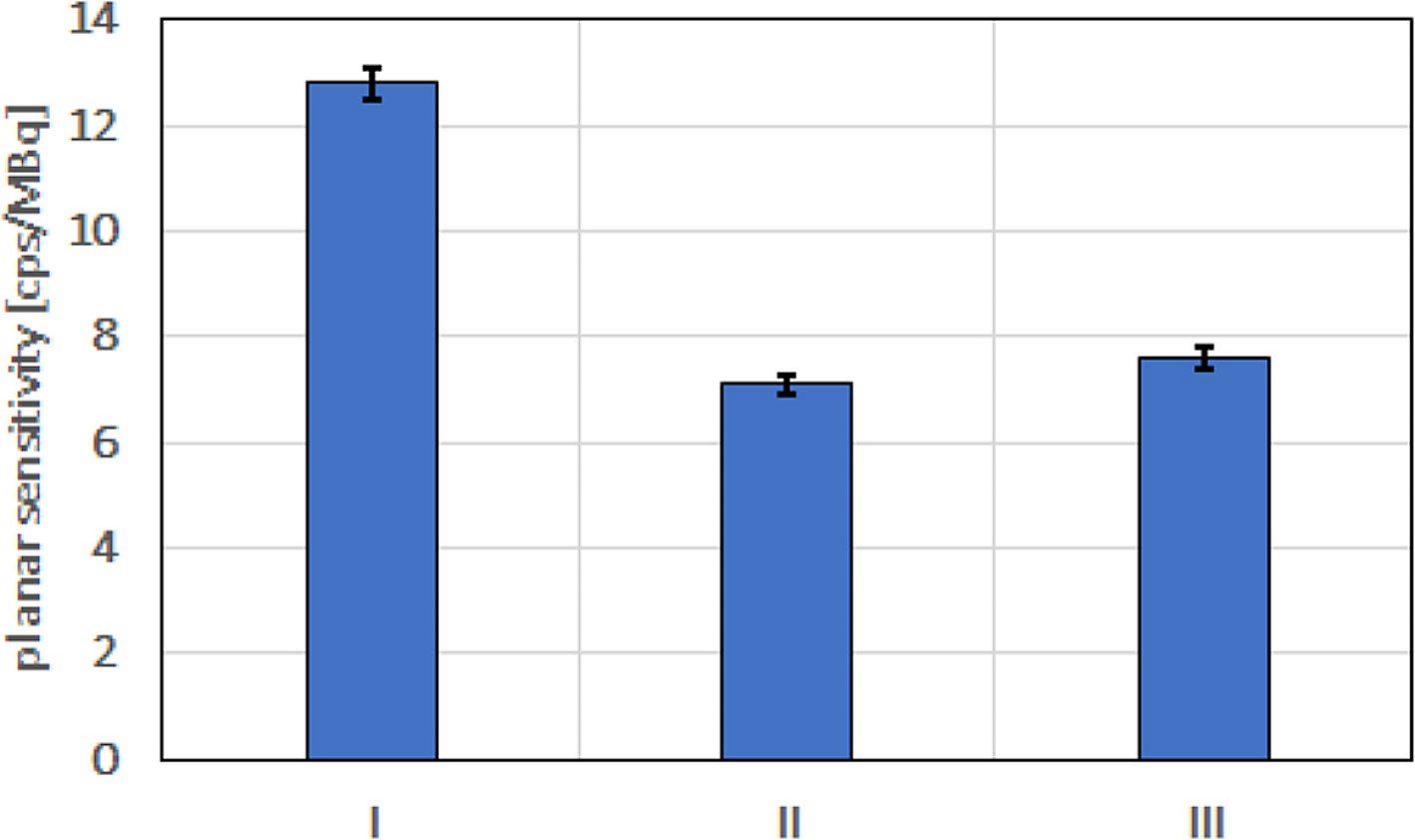
Planar sensitivities for ^225^Ac on the Symbia T6 gamma camera with HE collimators, and chosen main energy windows (I: 78 keV, width 20%, II: 217 keV, width 10%, III: 444 keV, width 10%)

The planar sensitivities measured for ^225^Ac on the Symbia T6 gamma camera with HE collimators and each main energy window are presented in Fig. 5.

**Fig. 6:**
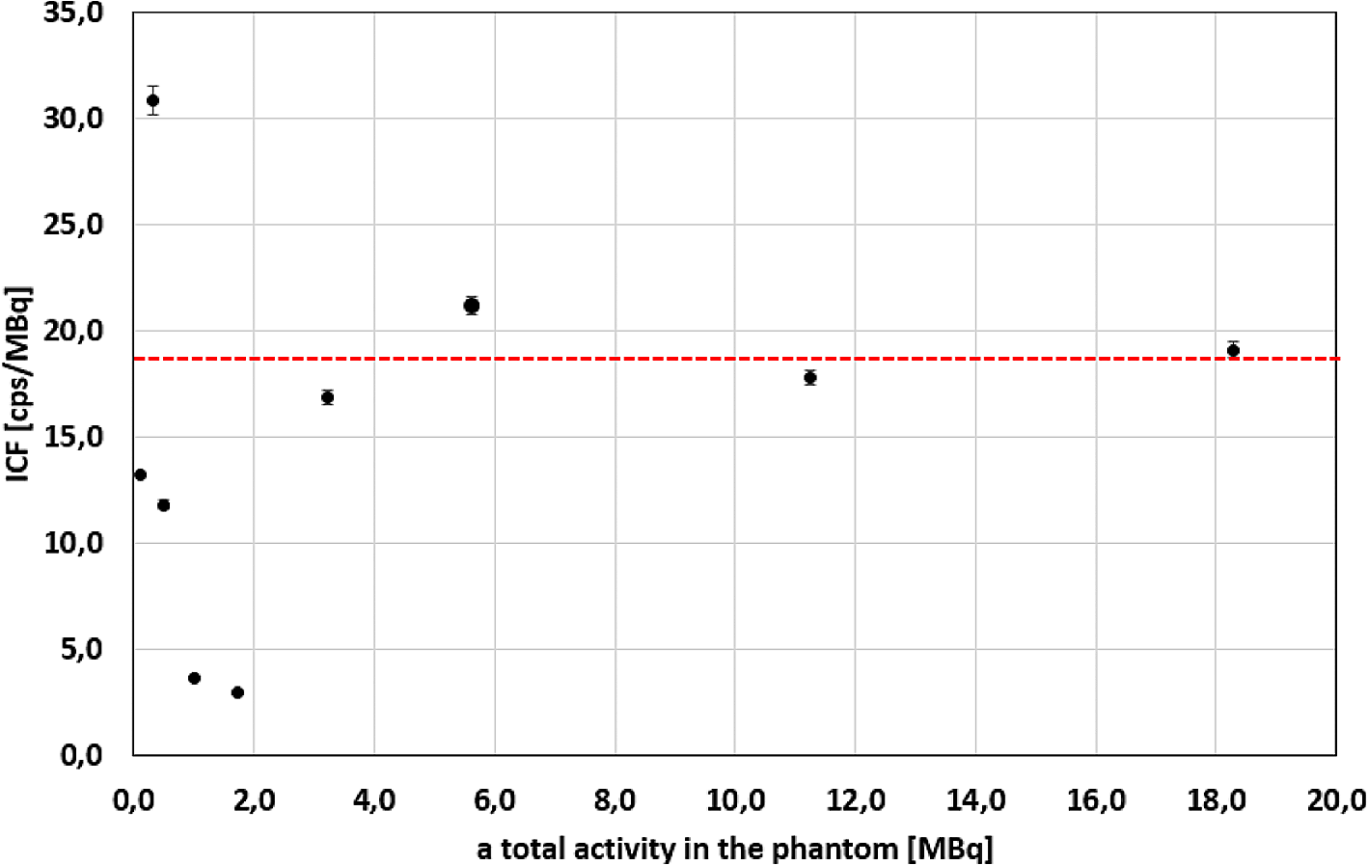
The imaging calibration factor for different values of total activity in the phantom. The red line shows an average ICF equal to (18.7 ± 1.8) cps/MBq, calculated for the first 4 data points

The imaging calibration factors calculated for all acquired images of the phantom are presented in Fig. 6. The ICFs stayed approximately constant (the percentage difference between the first ICF for a total activity equal to 18.3 MBq and other ICFs not more than 10%) for more than around 3 MBq of ^225^Ac. However, the ICF seemed to change randomly (increased and decreased) below 3 MBq. Due to this, only data points obtained with more than 3 MBq of total activity in the phantom were used to calculate the average ICFs. The average ICF calculated for 4 data points was (18.7 ± 1.8) cps/MBq. The value 1.8 cps/MBq was assumed to be the standard uncertainty of ICF (*u(ICF)*) for further calculations.

**Fig. 7:**
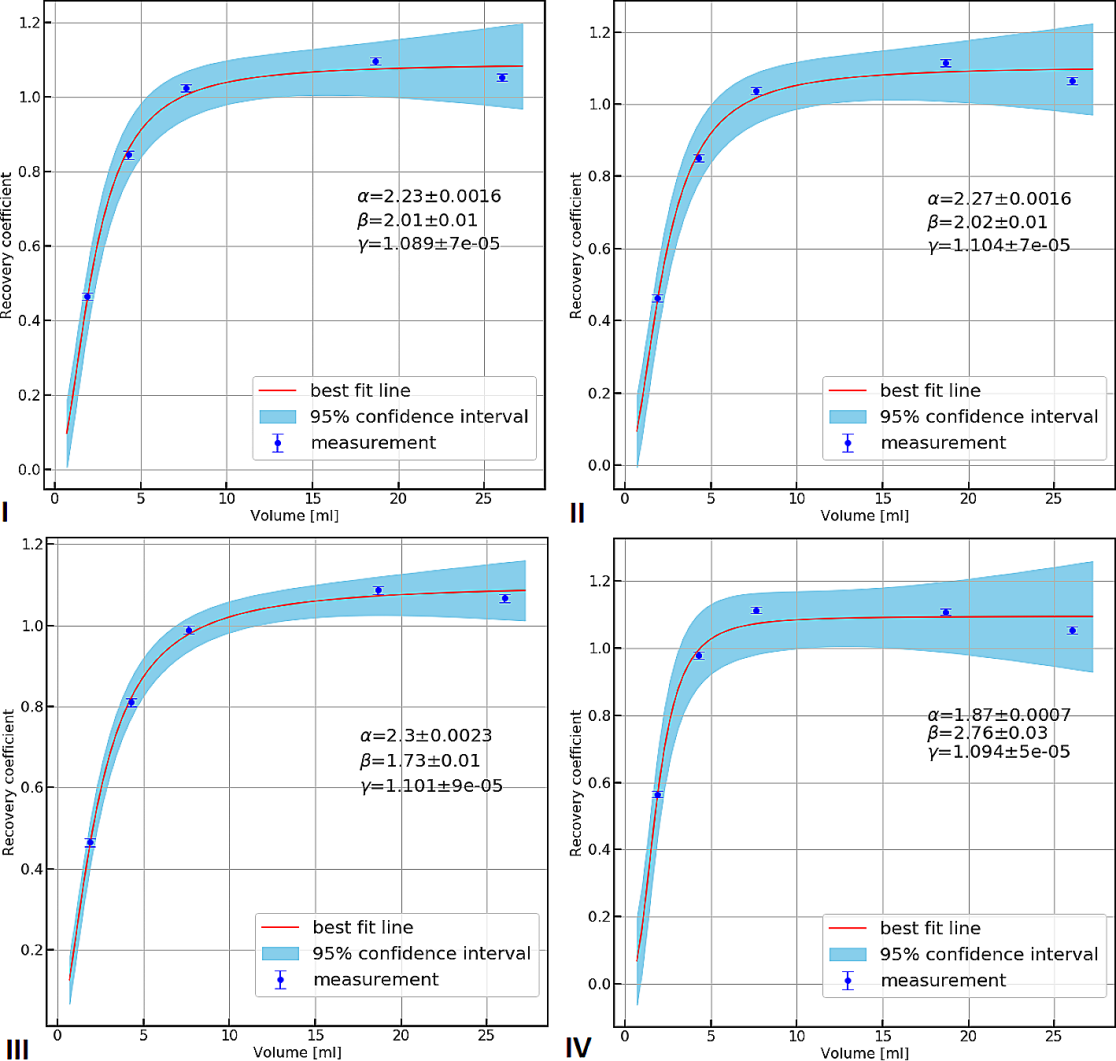
RCs and curve fits obtained with total activity in the phantom, I: 18.3 MBq, II: 11.3 MBq, III. 5.6 MBq, IV. 3.2 MBq

The recovery coefficient (RC) curves with the associated 95% confidence intervals, obtained for four images, for which the ICFs stayed approximately constant, are shown in Fig. 7. The curve fit parameters and the associated standard error (*SE*) are also presented in Table 3.

**Table 3:** The fitting parameters for the RC curves

Total activity in the phantom [MBq]	Parameter	Value	SE
18.3	α	2.23 mL	0.0016 mL
	β	2.01	0.01
	γ	1.089	7 × 10^− 5^
11.3	α	2.27 mL	0.0016 mL
	β	2.02	0.01
	γ	1.104	7 × 10^− 5^
5.6	α	2.3 mL	0.0023 mL
	β	1.73	0.01
	γ	1.101	9 × 10^− 5^
3.2	α	1.87 mL	0.0007 mL
	β	2.76	0.03
	γ	1.094	5 × 10^− 5^

**Fig. 8:**
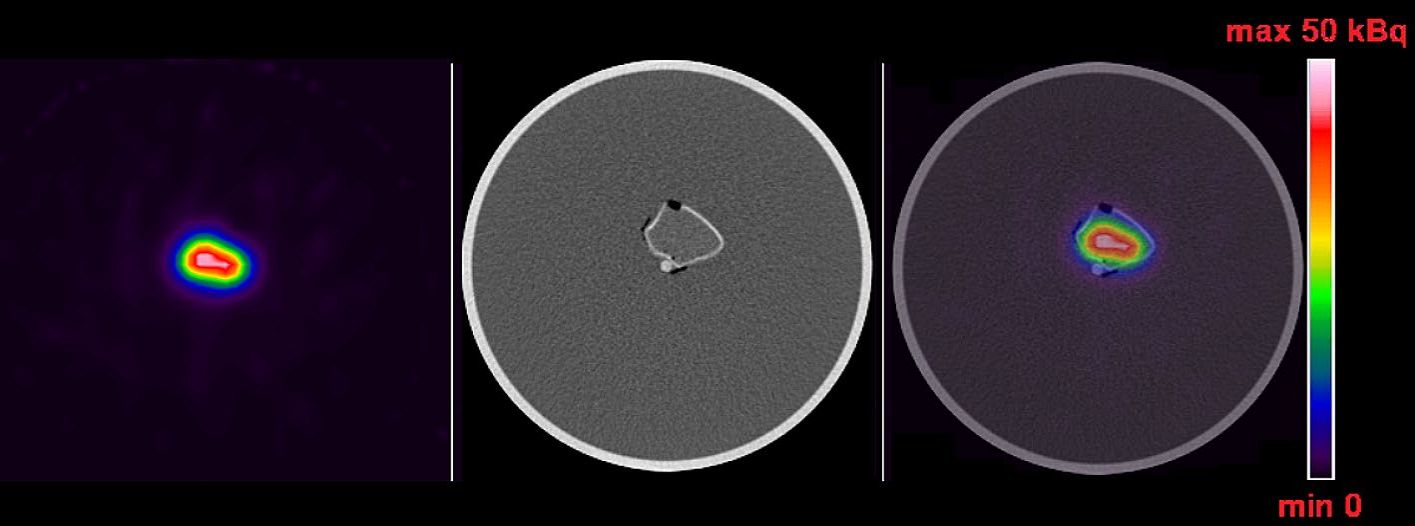
SPECT/CT image of the 3D phantom (SPECT, CT, fusion). The quantitative value on the color scale corresponds to the activity per voxel

Figure 8 shows SPECT/CT image of the 3D phantom. Table 4 summarizes the quantification of this image obtained by using the average ICF and data from the I RC curve (a total activity in the phantom of 18.3 MBq, with an activity concentration of 235 kBq/mL). The combined standard uncertainty of the calculated activity is also given.

**Table 4:** Comparison of the actual activity in the 3D phantom and the calculated activity using the proposed SPECT imaging protocol and calculation methodology

Actual activity in the 3D phantom [MBq]	A_3DPhantom_ [MBq]	Combined standard uncertainty A_3DPhantom_ [%]	%D
6.1 ± 0.1	6.4 ± 0.6	10.0	5.0

## Discussion

The study investigates and confirms the feasibility of quantitative SPECT/CT imaging of ^225^Ac with multiple energy windows, but reproduces specific conditions of TAT using substance P labelled with ^225^Ac isotope in the case of patients with relapsed glioblastoma. The proposed SPECT image protocol and calibration method demonstrate the possibility to quantify ^225^Ac activity in this specific case with an uncertainty of no more than 10% and satisfying accuracy. However, one should indicate that local treatment of glioblastoma is very specific condition, as the total therapeutic activity is concentrated in a relatively small volume, without any accumulating tissues in the surrounding. From the imaging perspective, this situation is different compared to the conditions during, e.g., [^225^Ac]Ac-PSMA or [^225^Ac]Ac-DOTATATE treatment, for which a similar amount of therapeutic activity is injected and distributes in the whole patient body, forming a rather complex uptake pattern.

There are some limitations of this study which, however, should be treated as further challenges to overcome.

The first limitation is related to the method of determining the ICF itself. In most quantitative studies, tomographic scans of large cylindrical phantoms without inserts filled uniformly with accurately measured activity have been used to calculate the imaging calibration factor. We used small containers filled with activity placed in a large cylinder filled with non-radioactive water (cold background). Zhao W et al. evaluated different calibration methods and compared their performance, for SPECT quantitation of therapeutic radioisotopes (^131^I, ^177^Lu, and ^188^Re) [21]. They found that camera calibration performed using tomographic scanning of the source(s) placed in the non-radioactive (cold) background can overestimate ICF by more than 10%., due to approximations made by the triple energy window scatter correction (TEW). Thus, they did not recommend the use of this method for the determination of the camera ICF for the studied isotopes. However, ^225^Ac is an isotope with limited availability at the moment and expensive to obtain, compared to therapeutic beta-emitting radionuclides. The annual global commercial supply of ^225^Ac is approximately 37 GBq [22]. For this reason, only a small amount of ^225^Ac activity is available for experimental studies and must be used efficiently. Therefore, we decided to use the same phantom to determine the ICF and RC(V) curves. On the other hand, the phantom prepared in this way reflects the exact and specific conditions of TAT using substance P labelled with ^225^Ac isotope in patients with recurrent glioblastoma. The last but not least argument is that we used quite a complicated acquisition protocol for ^225^Ac imaging (three main energy windows, three additional for SC). It is difficult to say whether the conclusions of Zhao W et al. are also binding in the case of such an acquisition [21].

The second limitation is related to the noise in reconstructed SPECT images used for ICF determination, and also R(V) curves. In our phantom experiment, we reproduced a selected clinical situation (activity only in cylindrical sources, no activity in the background). However, the apparent background signal in the images was significant, despite applying the described AC and SC corrections. It can be estimated that the background signal (total counts in the background) exceeded the signal from all cylindrical sources by more than twice in the image of the entire phantom obtained even for the highest activity concentration of ^225^Ac used in this study. A real background signal was only a part of the apparent background signal (image of the phantom without an activity; data not shown). The remaining background counts most probably came from septal penetration by high-energy photons, and also insufficient scatter correction taking into account only lower energy windows. For these reasons, we decided to subtract the apparent background signal from the total number of counts in the phantom before calculating the ICF. As a result, we obtained not only the determination of the ICF after taking into account the high background effect but also a better representation of the RC curves, which reached a plateau around the value of 1. However, as Kvassheim M et al. suggested, it may be inappropriate to force the RC function to 1 and because of this, a third fitting parameter should be added [23]. We added it in the form of *γ* parameter in Eq. 6. The gamma value was found to be around 1.1 for all calculations in our experiment. This most likely means that the simple background subtraction method we used to calculate the ICF works, but is not entirely accurate and depends on the choice of VOI position for determining the background counts. It could suggest that insufficient compensation of scatter or septal penetration lead to an object-dependent addition to the background signal. In principle, septal penetration and scatter should be adequately handled during quantitative SPECT reconstruction. It could be concluded that the DEW SC correction method used is not optimal for ^225^Ac quantitative imaging with three main energy windows, but it is the only one currently available in our department for this purpose. Appropriate algorithms are of limited availability in commercial software packages.

It is very limited data in the literature in discussed field. Our results can be compared with only a single report from the literature. Benabdallah N et al. presented a paper aimed at investigating the feasibility and limitations of ^225^Ac quantitative SPECT [17]. The planar sensitivity in three main energy windows for ^225^Ac was about 3–4 times lower in our study for the Symbia T6, than those obtained by Benabdallah N et al. (despite using probably the same procedure as described in our article but with only slightly different widths of the main energy windows). Furthermore, Benabdallah N et al. also performed the NEMA IEC PET Body Phantom SPECT/CT studies allowing them to adapt the volume-specific calibration factors (CF). They did not use SC correction during image acquisition with the Symbia T6, because according to them, the Esoft software does not allow scatter correction for multiple energy window acquisitions. We have to note that this is not true, because such protocol is available at this type of processing station. They found that CFs for ^225^Ac increase as the volume of the sphere increases, and reach a certain plateau quite quickly, which we observed in our experiment. This plateau was estimated at around 40 cps/MBq for the Discovery 670 and the Optima 640, and it was therefore surprising to see a plateau of around 10,000 cps/MBq for the Symbia T6. Nonetheless, the authors demonstrated on the 3D-printed model that the quantification was achievable with ^225^Ac SPECT image using multiple energy windows.

Our study is focused on precisely defined conditions. However, it would be beneficial to perform subsequent measurements with a varying energy window setting and other scatter correction methods. Especially for the lower photopeaks, significant downscatter from upper photopeaks has to be properly modelled, which is not adequately handled by dual-energy window scatter correction.

## Conclusions

In conclusion, this work demonstrates that it is feasible to perform quantitative ^225^Ac SPECT/CT imaging, acquired using 440 keV γ-coemission from^213^Bi, 218 keV γ-coemission from ^221^Fr, bremsstrahlung X-rays of ^209^Pb, and a gamma camera equipped with HE collimators. The proposed SPECT image protocol and the calibration method are presented. However, there are still many more challenges that should be considered for further research on this topic (among others seekingoptimal methods for SC correction).

## Data Availability

Freely available upon request.

## Abbreviations

α: Alpha
β: Beta
γ: Gamma
^225^Ac: Actinium-225
^213^Bi: Bismutium-213
^209^Bi: Bismutium-209
^221^Fr: Fransium-221
^217^At: Astatin-217
^213^Po: Polonium-213
^209^Tl: Thallium-209
^209^Pb: Lead-209
^133^Xe: Xenonium-133
^111^In: Indium-111
^18^F: Flourine-18
^177^Lu: Lutetium-177
^131^I: Iodine-131
^186^Re: Renium-186
^223^Ra: Radium-223
^227^Th: Thorium-227
SPECT: Single photon-emission computed tomography
CT: Computed tomography
PET: Positron Emission Tomography
AC: Attenuation correction
SC: Scatter correction
DEW: Dual energy window
TEW: Triple energy window
ICF: Imaging calibration factor
RC: Recovery coefficient
PSMA: Prostate-specific membrane antigen
DOTA: 2,2′,2”,2”’-(1,4,7,10-tetraazacyclododecane-1,4,7,10-tetrayl)tetraacetic acid
TAT: Targeted alpha therapy
HE: High energy
MELP: Medium energy low penetration
NEMA: National Electrical Manufacturers Association
IEC: International Electrotechnical Commission
ROI: Region of interest
VOI: Volume of interest
SE: Standard error
